# Mechanical and Tribological Properties of Porous Cu-15Ni-8Sn Alloy Fabricated Through Selective Laser Melting for Application in Self-Lubricating Bearing

**DOI:** 10.3390/ma18174197

**Published:** 2025-09-07

**Authors:** Hui Chen, Gengming Zhang, Jianling Liu, Shichao Liu

**Affiliations:** 1 School of Physics and Chemistry, Hunan First Normal University, Changsha 410205, China; aimeechenhui@163.com; 2 State Key Laboratory of Powder Metallurgy, Central South University, Changsha 410083, China; 216012@csu.edu.cn

**Keywords:** porous Cu-15Ni-8Sn, additive manufacturing, selective laser melting, self-lubricating bearing, tribological property

## Abstract

Additive manufacturing techniques, such as selective laser melting (SLM), enable the production of intricate and integrated components made from metallic materials with inherent porosity. The pores, typically perceived as defects, are commonly observed on the surface or within the matrix of SLM-formed components. However, it is noteworthy that these pores can function as reservoirs for lubricants to enhance tribological performance in specific applications, such as porous bearings. In this study, the optimum conditions for fabricating Cu-15Ni-8Sn alloy porous bearings via SLM technology were investigated. By regulating laser power and hatch space during SLM processing, Cu-15Ni-8Sn alloy porous bearings were successfully obtained. The resulting oil bearings exhibited an oil content exceeding 18% and a radial crushing strength surpassing 370 MPa. At reduced laser power (80 W) and increased hatch spacing (0.9 mm), average friction coefficients of 0.1 and 0.13 were observed, with volumetric wear values of 10.3 mm^3^ and 96.7 mm^3^, respectively. The friction mechanism is a combination of abrasive wear and delamination wear.

## 1. Introduction

Oil-impregnated porous metal bearings are extensively employed as self-lubricating components in aerospace, marine engineering, telecommunications, and transportation machinery [1,2]. These bearings are immersed in lubricants (e.g., lubricating oil) to saturate their pores and retain the lubricants [2,3], enabling autonomous lubrication during operation. Upon cessation of operation, the lubricant oil on the frictional surface is reabsorbed into the pores once again, minimizing wastage and extending operational life without requiring frequent replenishment.

Porous bearing materials are predominantly iron-based or copper-based, with copper-based variants accounting for approximately 35% [4,5]. Copper-based materials offer unique advantages over their iron-based counterparts, such as superior thermal conductivity, enhanced corrosion resistance, and non-magnetic permeability [6,7]. Menapace et al. successfully fabricated Cu-10 wt% Sn bronze bearings by mixing copper powder, prepared through atomization and electrolytic methods, with a certain proportion of tin powder [7]. Li et al. further improved Cu-10 wt% Sn oil-bearing materials by adding carbon nanotubes for surface modification [8]. The study found that an appropriate amount of carbon nanotubes dispersedly distributed in Cu-10 wt% Sn materials after sintering can improve mechanical properties and increase oil content, resulting in a certain level of lubrication effect. Apart from carbon nanotubes, graphite also has this effect. However, these studies provide limited insights into the friction behavior of oil-bearing surfaces.

Previous studies indicate that the addition of Ni to Cu-Sn binary alloys effectively reduces brittleness and enhances strength, attributed to the formation of newly generated intermetallic compounds (CuxNi1−x)3Sn [9]. Consequently, Cu-Ni-Sn ternary alloys are expected to replace Cu-Sn binary alloys as copper-based oil-bearing materials. In particular, the Cu-15Ni-8Sn alloy is considered a promising candidate for oil-bearing applications due to its exceptional resistance to thermal stress relaxation [10].

The fabrication of Cu-Ni-Sn ternary alloys has been achieved traditionally through powder metallurgy. In recent years, additive manufacturing (AM) techniques, such as selective laser melting (SLM), have gained significant attention for fabricating complex components. SLM, a powder-bed fusion technique, allows for the precise control of porosity, enabling the production of integrated, dimensionally complex structures without additional machining [11,12,13]. Foremostly, AM significantly reduces production time and allows for the fabrication of porous products without dimensional and structural constraints. Nevertheless, it is inevitable that the SLM-processed matrix contains a certain volume of pores due to residual air molecules inheriting their in situ position from raw powder particles [11,14]. While this porous nature is typically considered a disadvantage in traditional methods and targeted for elimination or mitigation in other studies, it serves as a natural advantage in preparing oil bearings within this study context. Zhu et al. employed SLM technology to prepare 316L stainless steel porous materials and controlled the porosity of designed parts by adjusting laser parameters [15], and their parts exhibited improved frictional performance with lubricant oil stored in pores. Hence, the SLM-fabricated Cu-15Ni-8Sn alloy porous components exhibit a combination of exceptional friction and wear resistance inherent to Cu-15Ni-8Sn alloys, along with the inherent oil lubrication capabilities of porous bearings [16,17].

This study investigates the influence of SLM processing parameters on the porosity of Cu-15Ni-8Sn alloy porous components while simultaneously characterizing their microstructure and evaluating their oil-bearing performance in terms of crushing strength and oil content. Additionally, friction and wear experiments are conducted to assess the frictional performance of bearings, providing insights into their potential for self-lubricating applications.

## 2. Materials and Methods

The SLM fabrication process employed pre-alloyed Cu-15Ni-8Sn-0.3Nb powders (D50 = 35 μm) to fabricate porous components. The chemical compositions of this pre-alloyed powder, analyzed using inductively coupled plasma (ICP) (ICAP 7000, Thermo Fisher, Waltham, MA, USA), revealed that Ni, Sn, and Nb contents accounted for 14.5%, 7.81%, and 0.28%, respectively, with the remaining composition being Cu. The experiments for fabricating porous components were conducted using SLM equipment (FS271M Farsoon, Farsoon High Tech, Changsha, Hunan, China) with variable parameters, as listed in Table 1, under a high-purity protection atmosphere with nitrogen gas at 99.99%. The laser scanning speed was set at a constant value of 400 mm/s, and the powder layer thickness was fixed at 30 μm. The powders were spread on the Cu substrate pre-heated to 100 °C, and the laser scanning strategy adopted an interlayer rotation angle of 67°. According to our previous work [18], the as-fabricated alloy is fcc single phase.

**Figure 1:**
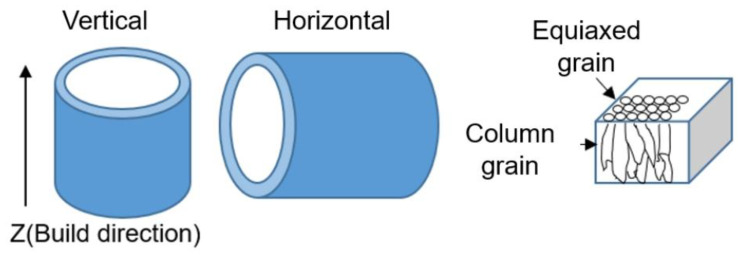
Schematic illustration of the fabrication process for vertical and horizontal porous Cu-15Ni-8Sn bearings, along with the typical microstructure obtained through selective laser melting (SLM).

The microstructure morphologies were observed using the DM4000M metalloscope (Leica, Wetzlar, Germany) and Quanta250 FEG (FEI, Portland, OR, USA) equipped with an energy-dispersive spectroscopy (EDS) system. The porosity of the porous bearing was roughly estimated by measuring the area occupied by pores in the surface morphology using Image J software (https://imagej.net/software/fiji/downloads accessed on 1 September 2025). Radial crushing strength and oil content are crucial performance indicators for oil-bearing applications. Radial crushing strength was determined by subjecting ring-shaped components to compression tests using an Instron 3369 test facility (Instron, Norwood, MA, USA), while oil content was measured by determining the volume of lubricant oil in the oil-immersed porous bearing through the Archimedes principle. Due to variations in components’ crushing directionality and microstructural differences between horizontal and vertical planes of SLM-ed components, samples with two different building orientations were prepared, as shown in Figure 1.

Friction and wear experiments were conducted on an MRH-1 friction and wear test machine (Jinan Yihua, Jinan, Shandong, China). Before testing, friction specimens were immersed in lubricant oil (Jet Oil II, Mobil, Spring, TX, USA) for 24 h. The dual friction rings utilized GCr15 special bearing steel with high hardness. The parameters for the frictional experiment were as follows: load of 500 N, rotation speed of 100 rpm, and friction time of 3600 s. For porous materials, a surface profile method was employed to measure the wear volume loss of the friction specimens before and after conducting frictional experiments using a VHX-5000 super depth-of-field 3D microscopy system (Keyence, Osaka, Japan). Details regarding pore characteristics were analyzed through mercury intrusion using AutoPore IV 9510 equipment (Micromeritics, Norcross, GA, USA).

## 3. Results

**Figure 2:**
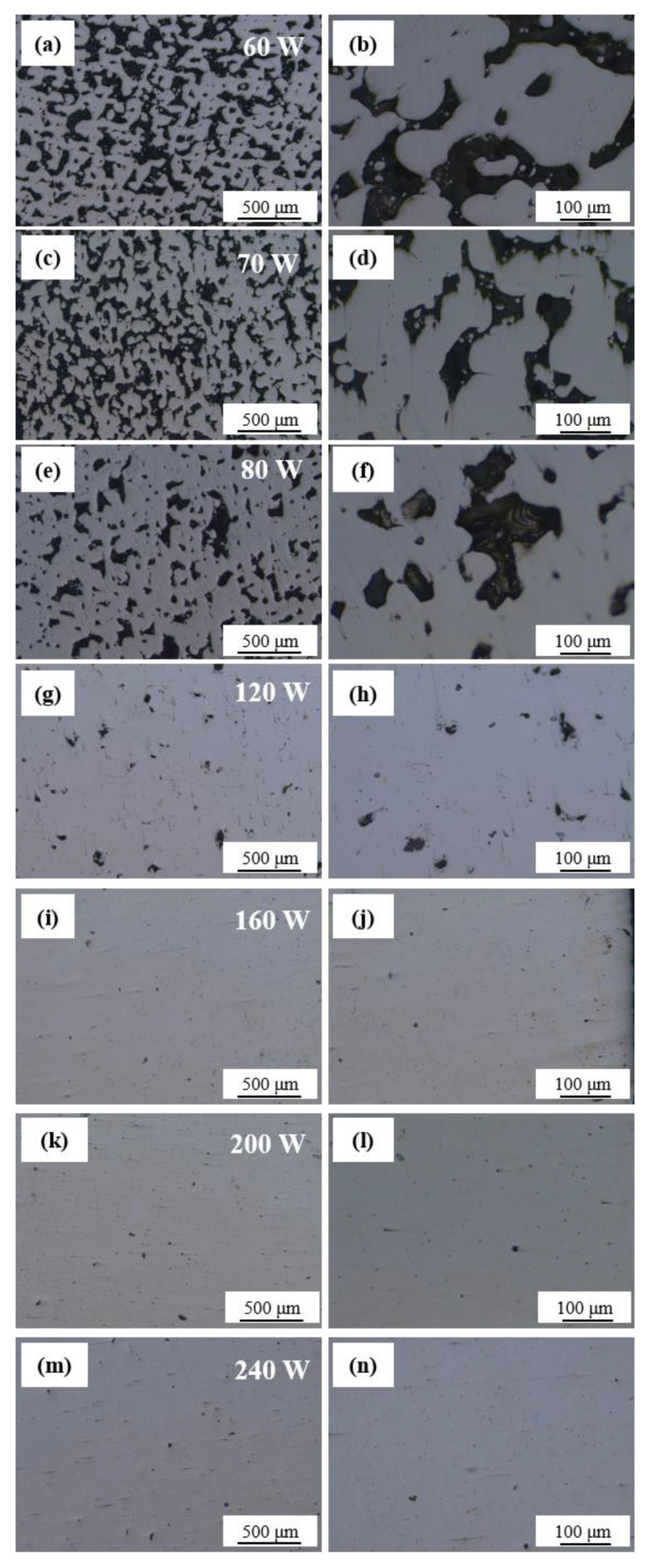
Microstructure of Cu-15Ni-8Sn alloy under different laser powers: (**a**,**b**) 60 W, (**c**,**d**) 70 W, (**e**,**f**) 80 W, (**g**,**h**) 120 W, (**i**,**j**) 160 W, (**k**,**l**) 200 W, (**m**,**n**) 240 W. The left (right)-hand images are at low (high) magnification.

Figure 2 shows the influence of laser power on the microstructure of porous components of Cu-15Ni-8Sn alloy. As the laser power increases, there is a decrease in both the size and quantity of pores. At a laser power of 60 W (Figure 2a,b), the pores exhibit an irregular shape, with sizes ranging from 60 to 200 μm, showing a relatively large variation range. When the laser power is increased to 70 W (Figure 2c,d), although the pores still maintain their irregular shape, their size ranges between 50 and 140 μm. Upon further increasing the laser power to 80 W (Figure 2e,f), the pore sizes are distributed within the range of 30 to 70 μm. With a further increase in laser power up to 240 W, both the size and quantity of pores in formed parts demonstrate a significant reduction trend (see Figure 2g–n). Higher laser powers lead to larger molten pool areas or longer-lasting heat-affected zones. The flow of molten material fills some pores at these higher powers, resulting in lower porosity, whereas a weaker flow occurs within smaller molten pools, leading to high porosity due to insufficient melting. However, it is noteworthy that for the laser powers of 160 W, 200 W, and 240 W, there are some precipitates in oval shapes, which are most probably copper oxides, which were generated during the oxidation induced by higher laser power [19,20].

**Figure 3:**
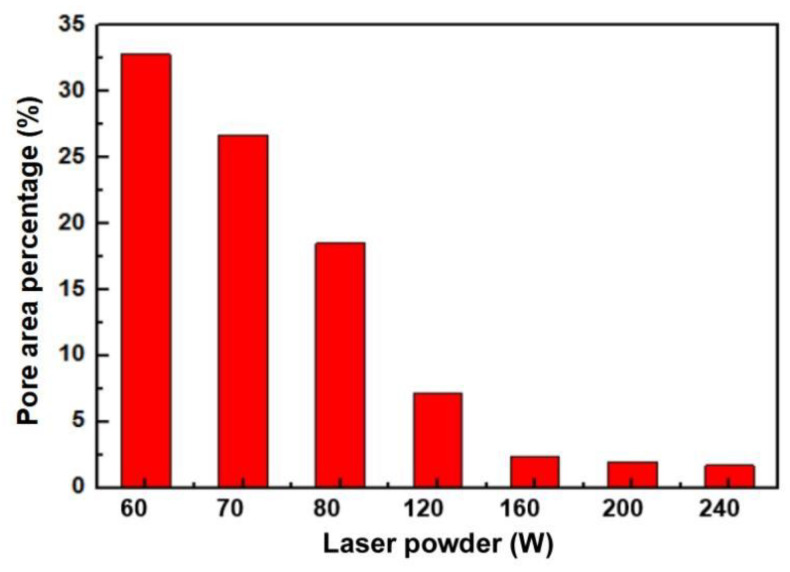
The average percentage of pore area of Cu-15Ni-8Sn alloy under different laser powders.

Statistical distribution calculations in Figure 3 reveal that when using a laser power of 60 W, pore area accounts for approximately 32.8% of the total matrix area. The percentage exhibits a significant decrease as the laser power is increased, ultimately reaching only approximately 1.7% at a laser power level of 240 W.

**Figure 4:**
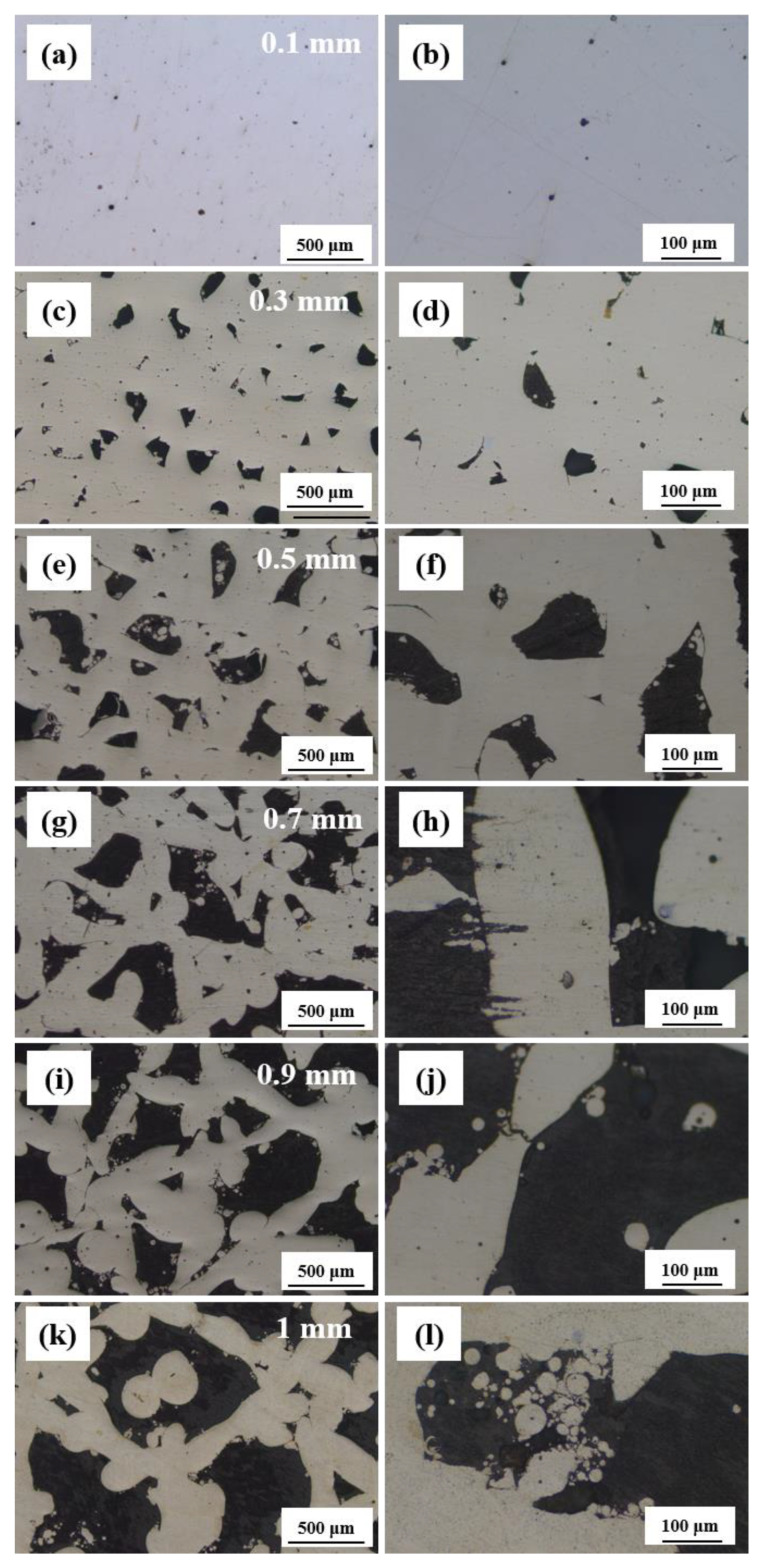
The microstructure of Cu-15Ni-8Sn alloy under different hatch spaces: (**a**,**b**) 0.1 mm, (**c**,**d**) 0.3 mm, (**e**,**f**) 0.5 mm, (**g**,**h**) 0.7 mm, (**i,j**) 0.9 mm, (**k**,**l**) 1 mm. The left (right)-hand images are at low (high) magnification.

**Figure 5:**
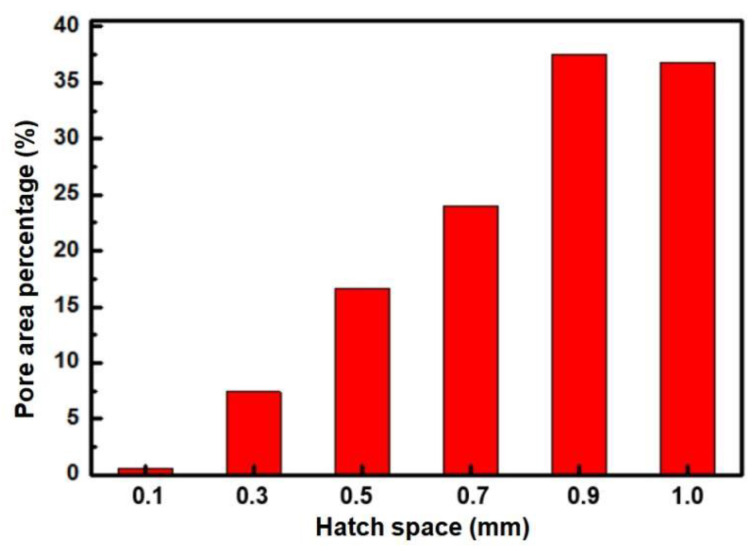
The average percentage of pore area of Cu-15Ni-8Sn alloy under different hatch spaces.

Figure 4 illustrates the influence of hatch space on the microstructure of Cu-15Ni-8Sn alloy. When the hatch space is 0.1 mm (Figure 4a,b), the matrix exhibits a few circular pores and homogeneous structure. With the further increase in hatch space, the quantity of pores increases, and the morphology becomes irregular (see Figure 4c–h). At a hatch space of 0.9 mm (Figure 4i,j), the corresponding oil content reaches 19.5%, and the matrix has a dentric structure. However, when the hatch space exceeds 1 mm (Figure 4k,l), there is a decrease in oil content due to the excessively large pores formed. The increase in hatch space results in reduced edge overlapping areas between melting tracks, leading to less heat absorption by the edges without re-melting and consequently causing sintered connections during the powder melting process. Therefore, an increase in porosity occurs with increasing hatch space, as shown by the statistical distribution calculation of pore areas presented in Figure 5. When the hatch space is 0.1 mm, the proportion of the pore area accounts for only 0.6%. However, increasing the hatch space to 0.9 mm significantly raises the percentage of the pore area to 37.4%. Further incrementing the hatch space to its maximum limit of 1 mm results in a slight reduction in the proportion of the pore area to 36.8%. Consequently, it can be observed that enlarging the hatch space leads to an enhanced overall proportion of the pore area.

**Figure 6:**
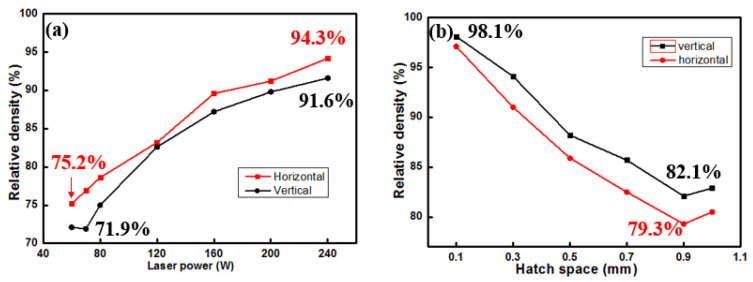
Effect of laser power (**a**) and hatch space (**b**) on relative density of Cu-15Ni-8Sn alloy.

The influence of parameters on the relative density of formed parts is illustrated in Figure 6. An increase in laser power leads to an enhancement of the relative density of formed components, while a larger hatch space results in a decrease in the relative density of formed components. The above-mentioned trends are the same in both vertical and horizontal orientations. In Figure 6a, the relative densities of horizontal orientations are a bit higher than the corresponding vertical samples. As for Figure 6b, the vertical orientations show slightly higher relative density than the horizontal orientations at different hatch spaces.

**Figure 7:**
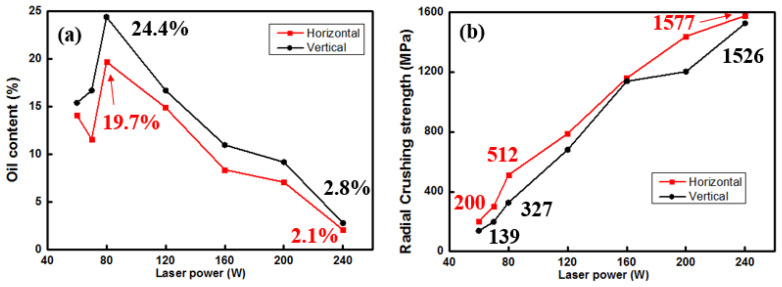
Effect of laser power on oil content (**a**) and radial crushing strength (**b**) of Cu-15Ni-8Sn porous bearings.

The variation trend of oil content is consistent with the change in porosity, as illustrated in Figure 7. At a laser power of 80 W, the optimal oil content of 24.4% is achieved for the vertical orientation. The vertically formed components show the same trend as the vertical, but all of the corresponding oil contents are lower. The radial crushing strength increases with the increasing laser power, aligning with the relative density trend and contrasting with the oil content trend. When the laser power is below 80 W, the oil content decreases instead of increasing due to large-sized pores being unable to retain lubricant oil effectively. There exist disparities in both oil content and radial crushing strength between longitudinally and transversely formed porous components produced via the SLM technique. Horizontally formed components exhibit relatively high radial crushing strength but comparatively low oil content owing to their formation being influenced by laser power.

**Figure 8:**
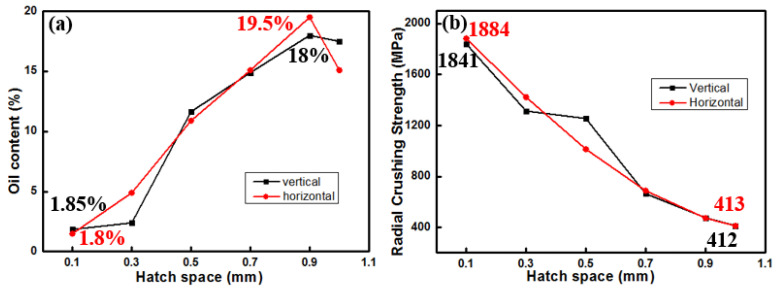
Effect of hatch space on oil content (**a**) and radial crushing strength (**b**) of Cu-15Ni-8Sn porous bearings.

The influence of hatch space on oil content and radial crushing strength is illustrated in Figure 8. When the hatch space increases to 0.9 mm, the oil content of the horizontally formed components reaches 19.5%, which is the highest (see Figure 8a). Within the range of hatch space from 0.1 to 1.0 mm, there is a decrease in the minimal radial crushing strength to approximately 412 MPa for vertically formed samples, which is almost the same as in the horizontal direction.

## 4. Discussion

**Figure 9:**
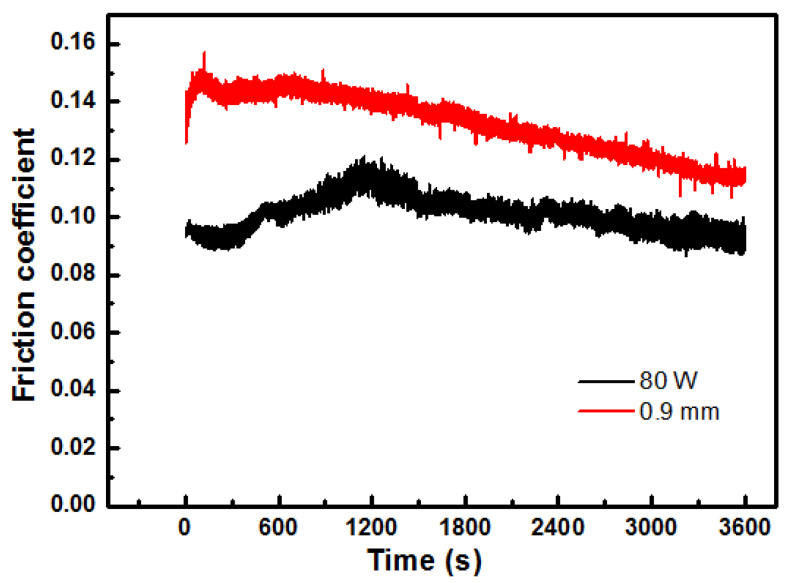
The relationship between friction coefficient and friction time.

Figure 9 displays the real-time friction coefficient of A3 and B5 samples. The initial friction coefficient is below 0.1, gradually increasing with the duration of friction. When the friction time is about 1200 s, the instantaneous friction coefficient reaches a peak value of 0.11, followed by a gradual and steady decline thereafter. Due to the high load (500 N) applied during the friction and wear test, the micro-convex bodies between the friction pairs become deeply embedded, preventing simultaneous storage of lubricating oil in their pores and resulting in a dry friction state initially. However, as the dry friction process progresses, these micro-convex bodies flatten out while generating frictional heat that causes expansion and overflow of lubricating oil from the pores of Cu-15Ni-8Sn alloy. Consequently, a lubricating film forms on the friction surface, exhibiting certain lubrication effects and transitioning the material into a boundary friction state with a subsequent decrease in the coefficient of friction. With further progression of the experiment, more lubricating oil expands and overflows due to generated heat between the surfaces undergoing friction. This leads to an increase in thickness of the formed lubricant film, gradually transforming it from a boundary to a mixed-friction state. The resultant mixed state encompasses both boundary and fluid frictions, leading to a further reduction in the coefficient of friction [4,21].

**Figure 10:**
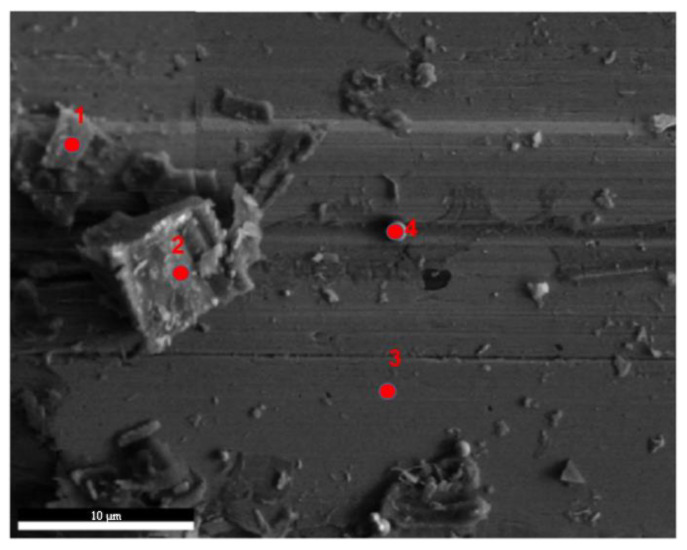
Surface SEM morphology of porous Cu-15Ni-8Sn sample after friction test (Point 1-4 are chosen for EDS analysis).

Figure 10 shows the surface SEM morphology of the porous Cu-15Ni-8Sn sample after the friction test. Continuous furrows were observed on the surface of the rubbed sample. EDS analysis results in Table 2 reveal that the predominant friction products contained Cu, Ni, Sn, and O elements. The high oxygen (O) content in points 1 and 2 indicates significant oxidation on the wear surface, with friction products primarily consisting of copper (Cu) and oxygen (O). Particularly at point 2, the atomic ratio of Cu to O is 1:1, suggesting that the friction product may be copper oxide (CuO). However, there is also a presence of chromium (Cr) element at point 1, which can be attributed to material transfer from the GCr15 steel counterpart during friction. The elemental composition at point 3 mainly comprises Cu, nickel (Ni), and tin (Sn). Point 4 represents spherical particles within a worn groove. Elemental energy spectrum analysis reveals a composition ratio similar to that of the matrix component at point 3, indicating that these particles are likely unmelted. In addition to continuous furrow distribution, surface peeling phenomena are observed on the worn surface, signifying a severe wear state for the Cu-15Ni-8Sn alloy [22,23].

In the severely worn state, the metal friction pair is actually experiencing boundary lubrication. The depth of the micro-convex body embedded in the friction pair significantly exceeds the thickness of the lubricating film on the friction surface. During the grinding process, the micro-convex body of hard GCr15 steel lacks an oil coating. Consequently, under high levels of frictional compressive stress and shear stress, it undergoes continuous scratching along the direction of frictional movement on the relatively soft Cu-15Ni-8Sn alloy surface, leading to the formation of furrows. As a result, grain wear occurs in the alloy material [24,25]. Moreover, as a result of elevated loads, the friction surface undergoes severe plastic deformation. The combined impact of continuous high-load frictional compressive stress and shear stress leads to significant accumulation of internal deformation stresses within both surfaces and subsurfaces. When these stresses surpass local thresholds for specific areas within alloys, cracks are initiated and subsequently propagate, ultimately resulting in material surface delamination [22]. In summary, under high load conditions, Cu-15Ni-8Sn alloy experiences a mixed mechanism consisting primarily of abrasive wear and delamination wear.

**Figure 11:**
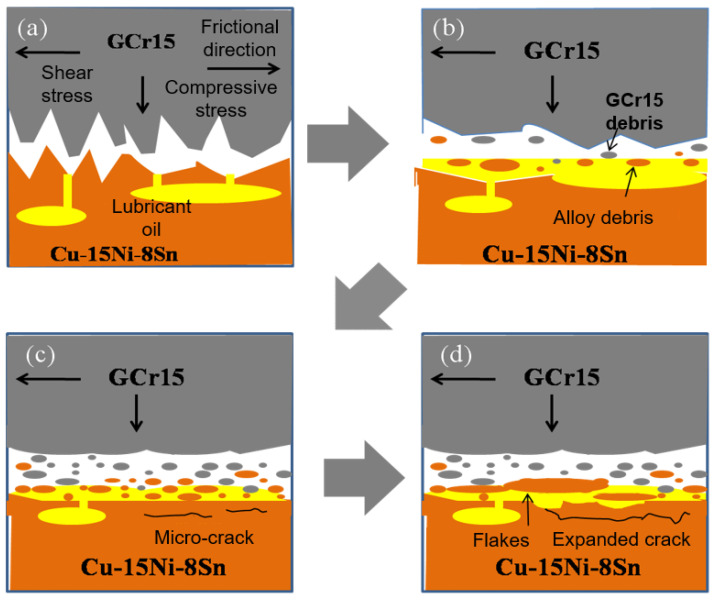
Schematic of the evolution on the worn surface of the SLMed porous bearing of Cu-15Ni-8Sn alloy in the frictional process: (**a**) friction begins, (**b**) abrasive wear, (**c**) small cracks appear on worn surfaces, (**d**) surface peeling.

During the process of friction, the friction surface layer of Cu-15Ni-8Sn alloy undergoes an evolutionary process, as illustrated in Figure 11. Due to the rigidity of GCr15 steel, the micro-convex bodies on the friction surface of Cu-15Ni-8Sn alloy experience plastic deformation and work hardening under the influence of frictional stress. Consequently, they fracture and generate fine abrasive debris, leading to abrasive wear (Figure 11b). Furthermore, thermal expansion during friction causes lubricant stored in pores to overflow and form a lubricant film between the friction interfaces at this stage, thereby enhancing friction performance. As friction progresses, the alloy’s friction surface gradually becomes smoother and transitions into a grinding process between its smooth surface and GCr15 steel’s micro-convex surface. The Cu-15Ni-8Sn alloy’s surface exhibits continuous furrow distribution along the direction of friction while under the action of frictive stress, causing micro-convexities on the surface of GCr15 steel to break into nanoparticles. Simultaneously, periodic frictive loads induce plastic deformation and accumulated internal stresses within the Cu-15Ni-8Sn alloy [26]. When these internal stresses exceed local limits for alloys’ internal stress levels, small cracks appear on worn surfaces (Figure 11c). At this time, there still exists a lubricating oil film that plays a certain role in providing lubrication during grinding between the alloy and GCr15 steel.

The lubricating oil stored in the pores undergoes thermal expansion, forming a lubricating film on the friction surface. However, due to the loss of the existing lubricating oil and the fact that there is no additional supplement, the lubricating film becomes thinner. Alloy wear leads to nucleation of micro-cracks on the subsurface, which gradually expand and connect with adjacent cracks. When these cracks reach the surface, surface peeling occurs [22,23,27], as shown in Figure 11d.

**Figure 12:**
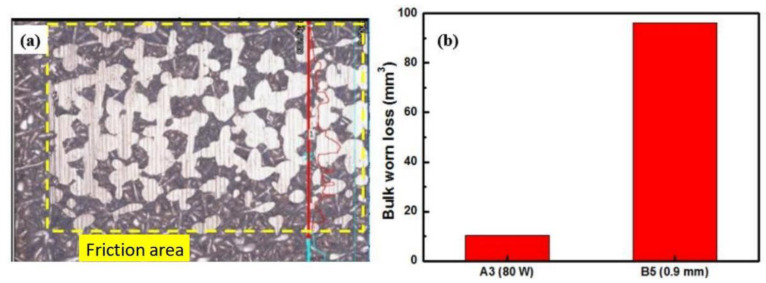
Morphologies of wear trace (**a**) and bulk worn loss (**b**) of Cu-15Ni-8Sn alloy.

After friction experiment, samples were observed and measured using a 3D ultra-depth field microscope. The observation area is indicated by the dashed box in Figure 12a. The measured volume wear of the sample is presented in Figure 12b. The results indicate that under A3 (80 W) conditions, the friction sample exhibits lower worn loss and friction coefficient compared to that under condition B5 (0.9 mm). Therefore, the porous sample prepared under A3 (80 W) conditions demonstrates superior wear resistance.

**Figure 13:**
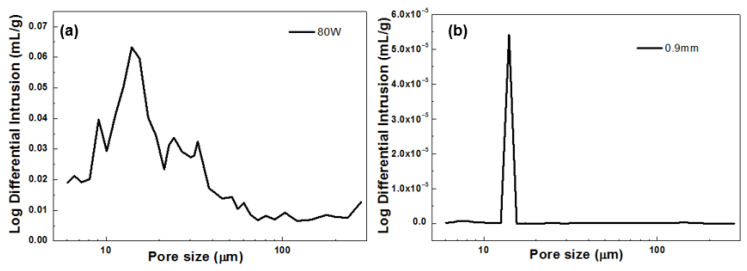
Pore diameter distribution curve of A3 (80 W) (**a**) and B5 (0.9 mm) (**b**).

The mercury intrusion method was performed to conduct pore analysis on the porous Cu-15Ni-8Sn specimen under A3 (80 W) and B5 (0.9 mm) conditions, and the pore size distribution curve shown in Figure 13 was obtained. Figure 13a shows that the pore size distribution of the A3 sample exhibits relatively high dispersion, with a majority falling within the range of 10–30 μm. Figure 13b reveals that the pore size distribution of sample B5 is more concentrated around 13 μm. It can be concluded that a pore size of 13 μm allows for maximum storage capacity of lubricating oil, which can appropriately overflow during frictional processes and form a lubricating film between the bearing and the shaft neck to enhance frictional behavior. These experimental findings are consistent with those presented in Figure 7, Figure 8 and Figure 9, confirming that the A3 (80 W) sample exhibits a lower friction coefficient.

## 5. Conclusions

In order to fabricate metal porous oil bearings using the selective laser melting (SLM) method, the influence of the SLM forming process on the porosity of the Cu-15Ni-8Sn alloy matrix was investigated by varying laser power or hatch space as independent variables. The main conclusions are as follows:

(1) The porosity of SLM-formed parts increases with the increase in scanning rate and initially increases and then decreases with the decrease in laser power. Cu-15Ni-8Sn alloy porous components were obtained by reducing the laser power or increasing the scanning distance.

(2) When the scanning rate is 400 mm/s and the layer thickness is 30 μm, using laser power of 80 W and hatch space of 0.2 mm, or using laser power of 340 W and hatch of 0.9 mm, porous components can be obtained with an oil content greater than 18% and radial crushing strength greater than 370 MPa, making them suitable for oil-bearing applications. The corresponding average friction coefficients are 0.1 and 0.12, while the volume wear amounts are measured at 10.3 mm^3^ and 96.7 mm^3^, respectively. The friction mechanism involves both abrasive wear and delamination wear.

(3) When the average size of Cu-15Ni-8Sn oil-bearing pores is approximately 13 μm, effective storage of lubricating oil and realization of the lubrication effect can be achieved during friction.

## Figures and Tables

**Table 1:** SLM parameters for Cu-15Ni-8Sn porous components.

No.	Laser Power (W)	Hatch Space (mm)	No.	Laser Power (W)	Hatch Space (mm)
A1	60	0.2	B1	340	0.1
A2	70	0.2	B2	340	0.3
A3	80	0.2	B3	340	0.5
A4	120	0.2	B4	340	0.7
A5	160	0.2	B5	340	0.9
A6	200	0.2	B6	340	1
A7	240	0.2	/	/	/

**Table 2:** EDS data measured at the marked locations 1–4 in Figure 10.

Elements	Point 1	Point 2	Point 3	Point 4
O (wt.%/at.%)	9.1/24.3	16.8/45.0	0.5/2.1	0.9/3.4
Nb (wt.%/at.%)	1.9/0.9	0.3/0.3	0.3/0.2	0.3/0.2
Sn (wt.%/at.%)	4.0/1.7	5.0/1.8	4.3/2.2	5.8/3.1
Cr (wt.%/at.%)	9.1/8.6	/	/	/
Fe (wt.%/at.%)	7.0/9.2	3.5/2.2	1.4/1.8	1.0/1.1
Ni (wt.%/at.%)	14.4/14.6	10.7/7.6	12.0/12.3	13.0/13.9
Cu (wt.%/at.%)	53.5/40.7	63.7/43.0	81.5/81.1	79.1/78.3

## Data Availability

The data presented in this study are available on request from the corresponding author due to another relevant research ongoing.
